# Impact of the Nitrogen on Nutrient Dynamics in Soybean–Grass Intercropping in a Degraded Pasture Area

**DOI:** 10.3390/plants14213372

**Published:** 2025-11-04

**Authors:** Karina Batista, Mayne Barboza Sarti, Laíze Aparecida Ferreira Vilela, Ricardo Alexander Peña Venegas, Gerardo Ojeda

**Affiliations:** 1 Instituto de Zootecnia—IZ, Agência Paulista de Tecnologia dos Agronegócios—APTA, 56 Heitor Penteado St. Centro, Nova Odessa 13380-011, SP, Brazil; mayneb.sartii@gmail.com; 2 Centro de Ciências da Natureza, Universidade Federal de São Carlos, Rodovia Lauri Simões de Barros, km 12—SP-189, Bairro Aracaçú, Buri 18290-000, SP, Brazil; laizevilela@gmail.com; 3 Agricultural Sciences and Production Department, Zamorano University, Box 93, Km. 30 Carretera de Tegucigalpa a Danlí, Valle del Yeguare, San Antonio de Oriente, Francisco Morazán P.O. Box 93, Honduras; rpena@zamorano.edu; 4 Escuela de Ciencias Agrícolas, Pecuarias y del Medio Ambiente ECAPMA, Universidad Nacional Abierta y a Distancia UNAD, Cl. 14 Sur # 14-23, Bogotá 111511, DC, Colombia; franklin.ojeda@unad.edu.co

**Keywords:** cropping system, legume, macronutrients, N use efficiency, sustainable agriculture, tropical forage

## Abstract

The development of an efficient agricultural system depends on the correct choice of crops and the management of nutrient supply and distribution within the system. This study aimed to determine how nitrogen (N) rates applied to rows of maize and tropical grass during the autumn–winter season (previous crop) influence subsequent intercropped plants. Treatments were arranged in a randomized complete block design with a split-plot scheme and four replications. The main plots comprised three cropping systems: soybean monoculture, soybean intercropped with Aruana Guinea grass (*Megathyrsus maximus* cv. Aruana), and soybean intercropped with Congo grass (*Urochloa ruziziensis* cv. Comum). The subplots consisted of four N rates (0, 50, 100, and 150 kg ha^−1^) applied to the rows of maize and tropical grass during the previous crop. Macronutrient accumulation and efficiency indices were determined for intercropped plants. Aruana Guinea grass increased the accumulation of N, phosphorus (P), potassium (K), and sulphur (S) in the soybean crop. N applied to the previous crop negatively affected the accumulation of P, K, and S in soybean monoculture. The maximum physiological efficiency of soybean was related to N supply. The efficiency indices for Aruana Guinea grass highlighted its ability to recover residual N applied to the previous crop.

## 1. Introduction

The recovery of degraded pasture areas offers a valuable opportunity to enhance food production and security [1]. Intercropping stands out as a promising approach among the strategies employed for recovering degraded pasture areas. Intercropping, defined as the simultaneous cultivation of two or more species within the same area and growing season, can be adapted to a wide range of environmental conditions and soil types [2]. However, the success of this system strongly depends on the appropriate selection of compatible species.

Tropical grasses in intercropping systems enhance soil cover, add biomass to soil organic matter, reduce erosion, and minimize water loss during dry periods. In addition, these grasses have deep roots that promote nutrient cycling and soil aggregation [3,4]. Meanwhile, legume plants in intercropping systems improve soil health by increasing soil organic matter and fixing atmospheric nitrogen. The legume plants also reduce the need for inorganic nitrogen fertilizers and improve nutrient use efficiency by 25–30% [5].

The synergistic combination between legume and grass in an intercropping system can promote beneficial systems, mainly with better nutrient use efficiency [6]. In an intercropping system, grass could absorb nitrogen (N) fixed by intercropped legumes and at the same time secrete root exudates to influence the N fixation of legumes. Moreover, the different root depths of the two crops lead to growth at different soil levels, reducing their competition. These would also lead to differences in nitrogen fixation efficiency in intercropping compared to monoculture [7].

The soybean–tropical grass intercropping system can be a promising alternative for recovering degraded pasture areas. The combination of these two species, with contrasting root systems and rhizosphere activity, can enhance soil coverage and improve N acquisition efficiency [8]. Additionally, this system can contribute to soil N enrichment through biological N fixation mediated by the *Rhizobium*–legume symbiosis [9]. In conditions of low soil N availability, as typically observed in degraded pasture areas, intercropping with legumes can reduce N stress in grasses while enhancing the N-fixing capacity of legumes [10]. Therefore, developing an effective N management strategy for a soybean–tropical grass intercropping system requires reliable indicators that capture the complexity of the N cycle and provide meaningful biological interpretation [11].

Climatic conditions influence crops’ growth and N demand, thereby directly affecting N use efficiency. Improving nitrogen use efficiency is therefore a key focus for sustainable agriculture, especially under changing climate conditions [12]. In intercropping systems, improvement of the overall N use efficiency can enhance the synchronization between soil N supply, applied N fertilizer, and crop N demand. Efficient N management is particularly crucial in areas of degraded pastures, where enhancing the N use efficiency can contribute to both soil restoration and increased productivity [13]. Nitrogen uptake efficiency can significantly increase due to the interaction of roots between intercropped species. Facilitation and competition coexist within legume–grass intercropping systems. Furthermore, the bi-directional nitrogen transfer and positive nitrogen competition among crops are beneficial for enhancing nitrogen use efficiency in the legume–grass relay intercropping system [14].

A study about nitrogen supply dynamics in maize–soybean intercropping identified mechanisms that drive efficient nitrogen uptake in this system [15]. Interspecific plant competition, where competition promotes the superior uptake of nitrogen by the maize. Root–microorganism interaction, in which the root system’s interaction with soil microorganisms enhances nutrient cycling and facilitates the transfer of N between plants, allowing the nitrogen fixed by the soybean to be absorbed and utilized by the maize. Enhanced nitrogen-fixing capacity by soybean, in that this crop increases nodulation, thereby boosting their own N-fixing capacity [15]. Therefore, the advantages of intercropping partially depend on below-ground interspecific plant interactions, which include interspecific facilitation, such as the complementary utilization of N resources and niche differences [5,7,14].

Crop N use efficiency is closely linked to the biomass accumulation and N allocation within the plant [16]. Macronutrient accumulation in intercropped species is influenced by inherent crop traits, photosynthetic efficiency, and environmental factors. Moreover, nutrient accumulation plays a central role in crop development, as intercropped species require a balanced nutrient supply to perform efficiently under competitive conditions. Therefore, evaluating the macronutrient accumulation patterns in soybean–tropical grass intercropping systems is essential for minimizing interspecific nutrient competition and optimizing performance in degraded pasture areas [17].

The results of a study about the effects of different nitrogen application methods on nitrogen use efficiency in a maize–soybean relay intercropping system, compared with monoculture plots, showed that the relay intercropping system significantly promoted N fertilizer utilization and resulted in a higher nitrogen recovery efficiency compared to the corresponding monocultures of maize and soybean [18]. A study about the global mean nitrogen use efficiency in croplands revealed that the heart of the problem is that current global mean nitrogen use efficiency is 48%. The authors concluded that through the implementation of optimal crop and fertilizer management strategies, this value could be substantially enhanced, with their models predicting a potential 30% increase (from 48% to 78%) [19].

The impact of maize nitrogen management on the subsequent soybean crop was investigated by the authors of [20], who concluded that the quantity of nitrogen fertilizer applied to the preceding maize crop did not have a substantial effect on the subsequent soybean crop. The authors proposed that only a large nitrogen surplus from over-fertilizing the maize crop could potentially limit the soybean crop, as it could reduce soybean nitrogen fixation more significantly than the gain in soil nitrogen supply. The findings of a study by the authors of [21] indicated that maize–soybean intercropping, when combined with optimal nitrogen fertilization, improved nitrogen content and total nitrogen uptake of the maize crop, thereby enhancing its nitrogen utilization efficiency indices such as nitrogen use efficiency, partial factor nitrogen use efficiency, nitrogen uptake efficiency, and nitrogen agronomic efficiency as compared with monocropping.

We hypothesized that N supplied to a preceding crop influences the performance of a subsequent cropping system. Therefore, this study aimed to determine how N rates applied to rows of maize and tropical grass during the autumn–winter season (previous crop) influence the subsequent soybean–tropical grass intercropping system in a degraded pasture area.

## 2. Results

### 2.1. Macronutrient Accumulations in Soybean

**Figure 1:**
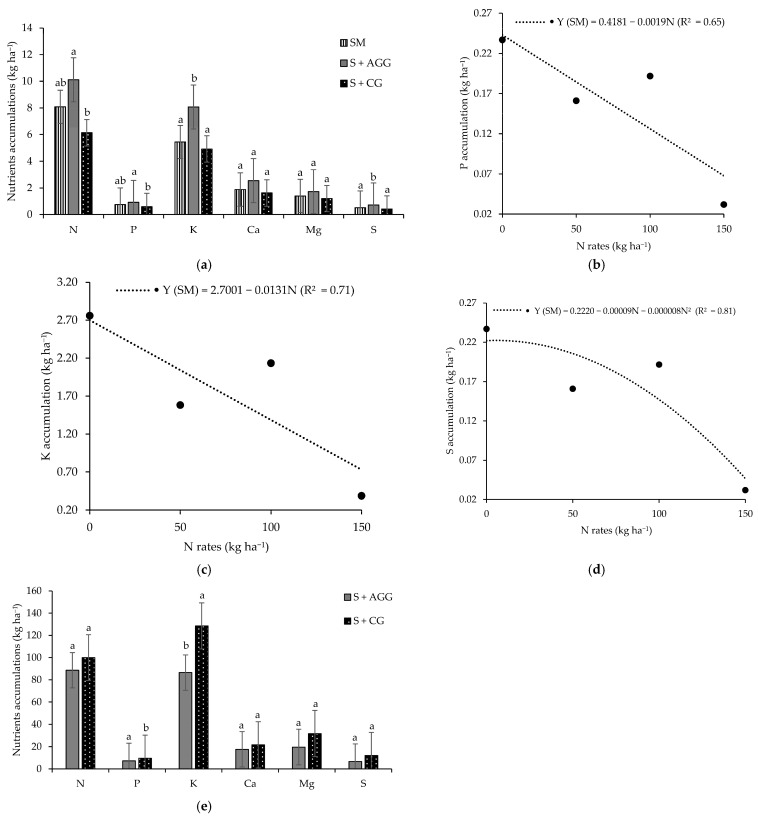
Macronutrients accum ulations in the soybean (**a**) as function of the cropping systems, accumulations of P (**b**), K (**c**), and S (**d**) in the soybean as function of the cropping systems and N rates applied to the previous crop and nutrients accumulations in the tropical grasses as function of the cropping systems (**e**). SM = soybean monoculture, S + AGG = soybean–Aruana Guinea grass intercropping, and S + CG = soybean–Congo grass intercropping. Means followed by different lowercase letter in the columns differ from each other according to Tukey’s test (*p* < 0.05).

In the first crop, soybean intercropped with Aruana Guinea grass exhibited higher accumulations of N, phosphorus (P), potassium (K), and sulphur (S) compared to other cropping systems (Figure 1a). Notably, the accumulations of K and S were approximately 1.6 times greater in this intercropping system than in the others. However, no significant differences were observed between soybean intercropped with Aruana Guinea grass and soybean monoculture for N and P accumulation. In contrast, the calcium (Ca) and magnesium (Mg) accumulation in soybean did not differ significantly among cropping systems during the first crop (Figure 1a).

In the second crop, N accumulation in soybean was not significant for the interaction between cropping systems and N rates applied to the previous crop, nor by either factor in isolation (Table 1). In contrast, the accumulations of P, K, Ca, Mg, and S in soybean showed significance for the interaction between cropping systems and N rates applied to the previous crop. Under monoculture conditions, P and K accumulations in soybean decreased linearly as the N rates increased (Figure 1a,c). The highest S accumulation (0.22 kg ha^−1^) in soybean monoculture was observed at an N rate of 5.63 kg ha^−1^ (Figure 1d). At an N rate of 50 kg ha^−1^, soybean intercropped with Congo grass had significantly greater accumulations of Ca, Mg, and S compared to accumulations in the soybean–Aruana Guinea grass intercropping system (Table 1).

### 2.2. Macronutrient Accumulations in the Grass

During the first crop, no significant differences were observed in the accumulation of N, P, Ca, Mg, or S in the grasses intercropped with soybean. However, K accumulation in the Congo grass was 1.5 times higher than that observed in the Aruana Guinea grass (Figure 1e).

**Figure 2:**
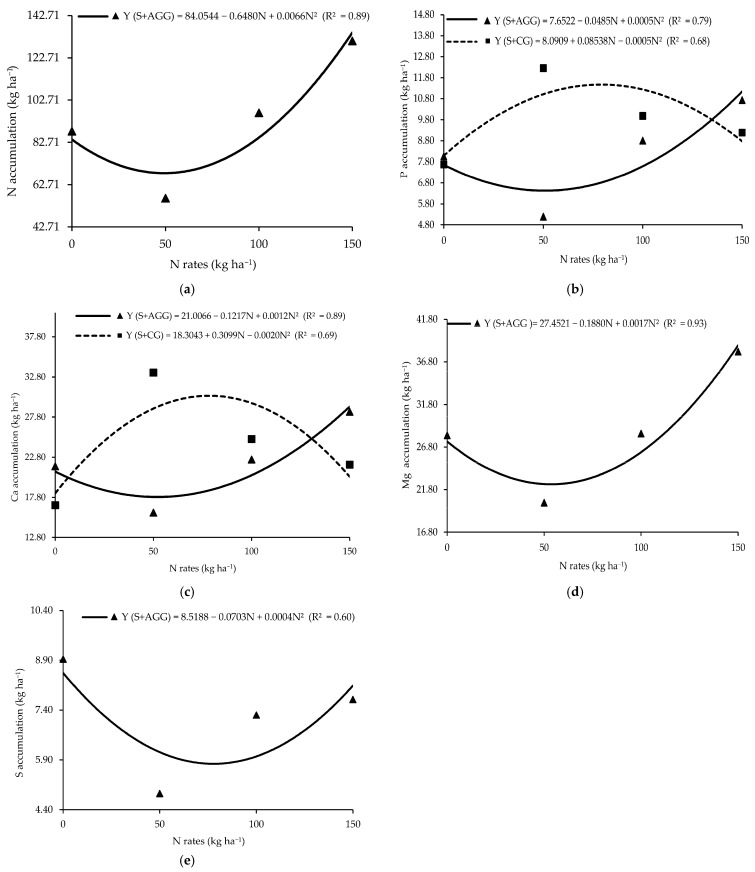
Accumulations of N (**a**), P (**b**), Ca (**c**), Mg (**d**), and S (**e**) in the tropical grasses as functions of the cropping systems and N rates applied to the previous crop. S + AGG = soybean–Aruana Guinea grass intercropping, and S + CG = soybean–Congo grass intercropping.

Macronutrient accumulation in the second crop showed a significant interaction between cropping system and N rate applied to the previous crop (Table 2). Aruana Guinea grass had the lowest N accumulation (68.15 kg ha^−1^) at an N rate of 49.09 kg ha^−1^ (Figure 2a). Similarly, the lowest P accumulation (6.48 kg ha^−1^) in Aruana Guinea grass occurred at an N rate of 48.5 kg ha^−1^ (Figure 2b), whereas the highest P accumulation (11.74 kg ha^−1^) was recorded in Congo grass at an N rate of 85.38 kg ha^−1^ (Figure 2b). The lowest Ca accumulation (17.92 kg ha^−1^) in Aruana Guinea grass occurred at 50.71 kg ha^−1^ of N, while the highest Ca accumulation (30.31 kg ha^−1^) in Congo grass was observed at an N rate of 77.48 kg ha^−1^ (Figure 2c). The minimum Mg accumulation in Aruana Guinea grass (22.25 kg ha^−1^) occurred at 55.29 kg ha^−1^ of N (Figure 2d). The lowest S accumulation (5.43 kg ha^−1^) in the Aruana Guinea grass occurred at an N rate of 87.88 kg ha^−1^ (Figure 2e). At an N rate of 50 kg ha^−1^, Congo grass accumulated significantly more P, K, Ca, Mg, and S than Aruana Guinea grass (Table 2).

### 2.3. Soybean Efficiency in Cropping Systems

**Figure 3:**
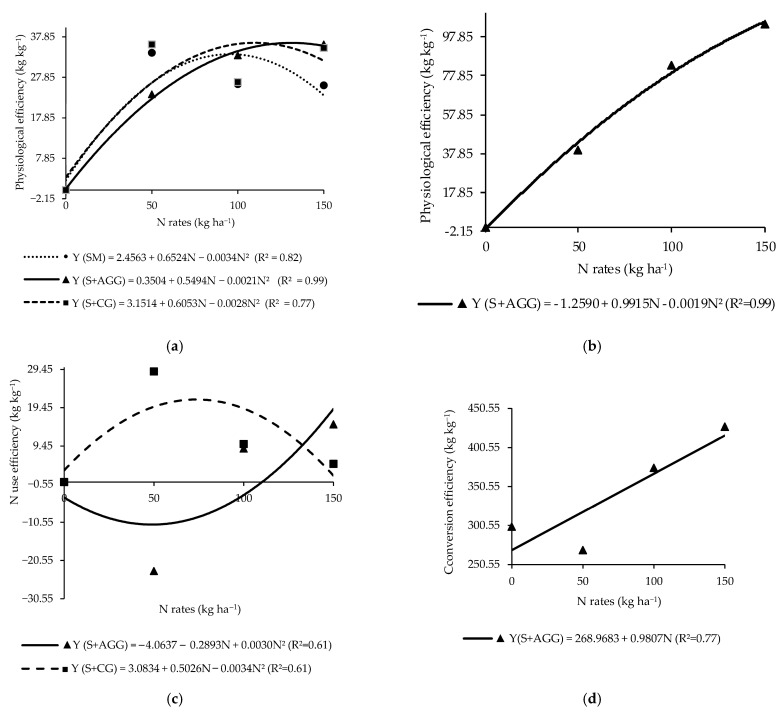
Physiological efficiency of the soybeans (**a**) and tropical grasses (**b**), N use efficiency of grasses (**c**), and conversion efficiency of N to biomass of tropical grasses (**d**) in the cropping systems as a function of the N rates applied to the previous crop. SM = soybean monoculture, S + AGG = soybean–Aruana Guinea grass intercropping, and S + CG = soybean–Congo grass intercropping.

The physiological efficiency of the soybean in the second crop showed a significant interaction between cropping systems and N rates applied to the previous crop (Table 3). In the soybean monoculture, the highest physiological efficiency (33.75 kg kg^−1^) was observed at an N rate of 95.94 kg ha^−1^ (Figure 3a). In the intercropping systems, soybean intercropped with Aruana Guinea grass had the highest physiological efficiency of 36.28 kg kg^−1^ at 130.81 kg ha^−1^ of N, while soybean intercropped with Congo grass reached a maximum value (35.86 kg kg^−1^) at 108.09 kg ha^−1^ of N (Figure 3a).

The N use efficiency differed significantly among cropping systems (Table 3). Soybean intercropped with Congo grass presented the highest N use efficiency, although it was not significantly different from soybean intercropped with Aruana Guinea grass (Table 3). In contrast, soybean monoculture had a negative N use efficiency, indicating inefficient utilization of the applied N to the previous in this system (Table 3).

The efficiency of conversion of N to biomass in soybean showed a significant interaction between cropping systems and N rates applied to the previous crop (Table 3). At the N rate of 50 kg ha^−1^, no significant difference was observed between soybean monoculture and soybean intercropped with Aruana Guinea grass (Table 3). However, soybean intercropped with Congo grass exhibited the highest efficiency of conversion of N to biomass at this rate. Moreover, across all N rates applied to the previous crop, soybean intercropped with Congo grass consistently showed the highest efficiency of conversion of N to biomass among the evaluated systems (Table 3).

### 2.4. Grasses Efficiency in Cropping Systems

The physiological efficiency, N use efficiency, and efficiency of conversion of N to biomass in the grasses showed significance for the interaction between cropping systems and N rates applied to the previous crop (Table 4).

The highest physiological efficiency (128.09 kg kg^−1^) for Aruana Guinea grass intercropped with soybean was estimated at an N rate beyond the range evaluated in this study (260.92 kg ha^−1^) (Figure 3b).

The maximum N use efficiency (21.66 kg kg^−1^) for Congo grass occurred at an N rate of 73.91 kg ha^−1^ (Figure 3c), whereas, the lowest N use efficiency (−11.04 kg kg^−1^) was observed in Aruana Guinea grass at an N rate of 48.22 kg ha^−1^ (Figure 3c). At an N rate of 50 kg ha^−1^, Congo grass showed significantly higher N use efficiency compared to Aruana Guinea grass (Table 4). The efficiency of N conversion to biomass in Aruana Guinea grass increased linearly with increasing N rates applied to the previous crop (Figure 3d). This finding indicated that this grass is N demanding.

## 3. Discussion

The increased accumulation of N, P, K, and S observed in soybean intercropped with Aruana Guinea grass during the first crop indicates that this grass did not compete with soybean and its presence promoted a beneficial effect in the uptake (Figure 1a). Beneficial interactions occur when one plant improves the environmental conditions for another, typically via mycorrhizal associations or the release of root exudates and extracellular enzymes that increase nutrient availability [14,22].

The absence of significant effects on N accumulation in soybean, as influenced by the cropping system and N fertilization of the previous crop, may be due to an imbalance between soil N availability and plant demand (Table 1). High soil N availability is known to suppress biological nitrogen fixation and alter N uptake dynamics [23]. Furthermore, the authors of [24] highlighted that applied N is partitioned between plant uptake and immobilization (microbial or plant-mediated). The N taken up is accumulated in plant roots and shoots, while the remaining soil N can be potentially immobilized by soil microbes or through chemical associations in the soil.

In both cropping seasons, the macronutrient accumulation pattern in the soybean followed the order N > K > Ca > Mg > P > S (Figure 1 and Table 1). These findings are consistent with those observed by the authors of [23], regardless of water or N supply.

The reductions observed in P, K, and S accumulation in the soybean monoculture in the second crop suggest that the excessive N input in a crop can lead to nutrient imbalances in subsequent crops (Figure 1b–d). Balanced proportions of N, P, and K are essential for optimal plant development, and deviations from this balance can alter plant growth cycles and productivity [25]. The smallest K accumulation in Aruana Guinea grass during the first crop (Figure 2e), coupled with higher K accumulation in intercropped soybean, supports the hypothesis of reduced competition from this grass species. K is highly mobile in plant tissues and prone to leaching due to its lack of structural binding [26], which may explain its greater availability to soybean in this system.

The increased N accumulation in Aruana Guinea grass in response to higher N supply suggests an important role of this grass in N cycling, particularly under conditions of excess N (Figure 3a). Moreover, it highlights its potential to reduce N losses and enhance sustainability by scavenging N from deeper soil layers and making it available to subsequent crops [27].

The accumulation of P in the Aruana Guinea and Congo grasses showed a direct relationship with N supply to the previous crop (Figure 2b). Our findings suggest that Congo grass and Aruana Guinea grass intercropped with soybean exploit soil resources differently [28], with Aruana Guinea grass benefiting at a lower N rate than Congo grass. Differential P accumulation between Aruana Guinea and Congo grasses further suggests complementary strategies, where each species accesses distinct P pools in soil [29].

The highest accumulation of P, K, Ca, Mg, and S in Congo grass and soybean at the N rate of 50 kg ha^−1^ (Table 2) could be an effect of the root exudates of these plants [8]. Root exudates can alter rhizosphere chemistry and increase nutrient bioavailability, especially in intercropped systems [30].

Given the close relationship between N and S in amino acids synthesis, the reduced S accumulation under high N supply (87.88 kg ha^−1^) may cause an imbalanced N–S ratio (Figure 2e). Reference [31] noted that soil organic matter is a major S source for plants; thus, the use of S-rich residues can enhance S availability via mineralization.

The response of the physiological efficiency of the soybean monoculture to the N applied to the previous crop did not reflect the N accumulation in the soybean in this system (Figure 3a and Table 1). In contrast, intercropped systems appeared to modify N dynamics, leading to higher physiological efficiency (Figure 3a and Table 4). Reference [14] reported that the spatial distribution of roots in intercropping systems could influence plant physiological responses, with legumes typically exploring upper soil layers and grasses accessing deeper soil layers.

The negative N use efficiency in the soybean monoculture suggests higher N losses in this system (Table 3). Conversely, intercropping with Congo grass appeared to enhance the N use efficiency of soybean, supporting greater N cycling and uptake by soybean (Table 3). Reference [24] observed similar results and emphasized the importance of recycled N in intercropping systems. According to the authors of [32], crop N requirements are closely linked to N use efficiency.

The superior N use efficiency in Congo grass intercropped with soybean points to a complementary relationship between the two species (Table 4). According to the authors of [22], complementary root interactions can significantly enhance N uptake and utilization. Root interactions, N transfer, and positive competition between species can improve N recovery and absorption efficiency [14,33].

The low N use efficiency in Aruana Guinea grass is aligned with its low N accumulation (Table 4 and Figure 3c). However, its conversion efficiency was influenced by N availability (Figure 3d), suggesting that its biomass residues could play a role in N cycling and loss reduction under high-N conditions during the dry season [24]. Intercropping is a promising practice to improve N efficiency through ecological resource use [14,33].

The lack of response to N rates in the efficiency of the conversion of N to biomass of Congo grass (Table 4) suggests that this grass may have utilized excess N released by the soybean nodules. Differences in crop morphology and photosynthetic capacity contribute to varied N requirements. In legumes such as soybean, nodulation is closely associated with growth and surplus N is released by the nodules when plant demand is exceeded [32].

## 4. Materials and Methods

### 4.1. Characterization of the Experimental Site

**Figure 4:**
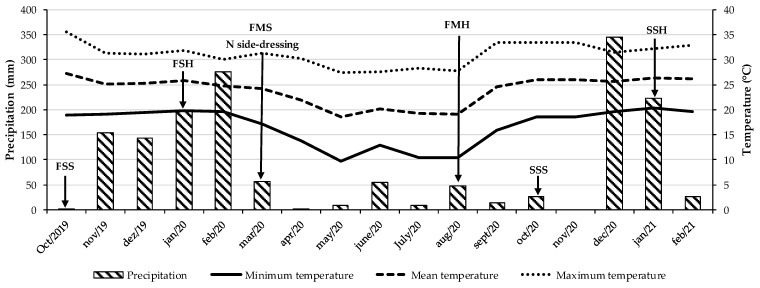
Precipitation and temperature data during the experimental period. FSS: first soybean sowing. FHS: first soybean harvest. FMS: first maize sowing, N side-dressing: N rates applied manually as side-dressing to the rows of maize and tropical grass during the autumn–winter season (the previous crop). FMH: first maize harvest. SSS: second soybean sowing. SSH: second soybean harvest.

The experiment was carried out in southeastern Brazil (22°42′ S, 47°18′ W, and 570 m altitude) in a red-yellow argisol [34] classified as an ultisol according to the United States Department of Agriculture soil taxonomy (USDA) [35] from August 2019 to September 2021. According to the Köppen climate classification, the region’s climate is type Aw—characterized by a tropical savanna climate with a marked rainy season in summer and a dry season in winter [36]. Figure 4 presents the precipitation and temperature data recorded throughout the experimental period.

The experimental field was a severely degraded pasture before this study. Before the experiment, a Dutch auger (Sondaterra^®^ TP-3”, Piracicaba, São Paulo, Brazil) was used to collect soil samples at a depth of 0–20 cm for chemical and physical characterization. The chemical analyses of the soil revealed the following values: pH (CaCl_2_) = 4.7; organic matter (colorimetric method) = 30 g dm^−3^; phosphorus (resin) = 4 mg dm^−3^; potassium (resin) = 1.5 mmolc dm^−3^; calcium (resin) = 10.0 mmolc dm^−3^; magnesium (resin) = 7.0 mmolc dm^−3^; potential acidity (H + Al, SMP buffer solution method) = 47 mmolc dm^−3^; sulfate (SO_4_^−2^, turbidimetric method) = 9.0 mg dm^−3^; sum of extractable bases = 19.0 mmol_c_ dm^−3^; cation exchange capacity = 66.00 mmol_c_ dm^−3^; and base saturation = 28%. The physical analyses showed clay (<0.002 mm) = 239 g kg^−1^, silt (0.053–0.002 mm) = 91 g kg^−1^, total sand = 670 g kg^−1^, coarse sand (2.00–0.210 mm) = 120 g kg^−1^, and fine sand (0.210–0.053 mm) = 550 g kg^−1^. According to the USDA Soil Texture Calculator, the soil was classified as sandy clay loam [35]. The soil analysis methods were as follows: the colorimetric method for organic matter content [37]; the ion-exchange resin procedure for P resin, K resin, Ca resin, and Mg resin [38]; the SMP buffer solution method for H + Al [39]; the turbidimetric method for SO_4_ [40,41]; and base saturation (BS%) calculated using the formula in [42].

During the experimental period, maize–tropical grass intercropping received nitrogen at different rates in the autumn–winter (previous crop), followed by soybean intercropped with tropical grass in the summer. This paper presents results from two soybean cropping seasons: 2019/2020 (first crop) and 2020/2021 (second crop). The soybean crop was harvested for ensiling at the R7 stage, which was when the plants were beginning to mature and the pods had reached mature colour on the main stem.

### 4.2. Experimental Design

The treatments were arranged in a randomized complete block design with a split-plot scheme and four replications. The main plots comprised three cropping systems: (1) soybean monoculture; (2) soybean intercropped with Aruana Guinea grass (*Megathyrsus maximus* cv. Aruana); (3) soybean intercropped with Congo grass (*Urochloa ruziziensis* cv. Comum). The subplots consisted of four N rates (0, 50, 100, and 150 kg ha^−1^), which were applied manually as side-dressing in the rows of maize and tropical grass during the autumn–winter season (the previous crop). This application was performed when maize plants had 5–6 fully expanded leaves, corresponding to the V5–V6 growth stage. The effects of the N rates on soybeans were evaluated only in the second crop. Thus, the treatments in the first crop were only the cropping systems, (1) soybean monoculture, (2) soybean intercropped with Aruana Guinea grass, and (3) soybean intercropped with Congo grass, in a randomized complete block design.

### 4.3. Field Management and Details

Prior to planting the first soybean crop, the soil was ploughed, harrowed, and limed. According to soil chemistry, 2 t ha^−1^ of dolomitic limestone (>12% MgO) and 72 kg ha^−1^ of P_2_O_5_ were applied. Fertilization at planting was applied only to soybean rows, at rates of 17 kg ha^−1^ N, 59 kg ha^−1^ P_2_O_5_, and 34 kg ha^−1^ K_2_O [43].

The soybean cultivar used was M6410IPRO (INTACTA RR2 PRO^®^, Agro Bayer Brazil), which was inoculated with *Bradyrhizobium japonicum* at the planting time. Soybean rows were spaced 0.45 m apart, with a plant density of 300,000 plants per hectare. In the intercropping systems, soybean and tropical grass were sown simultaneously using a sowing–fertilizer machine equipped with separate dispenser boxes for large and small seeds. In these systems, grass rows were intercalated between soybean rows at a spacing of 0.225 m apart. Grass seeds with a cultural value of 60% were sown at a rate of 6kg ha^−1^. Experimental plots measured 72 m^2^ (3.6 m × 20 m).

The biomass (soybean or soybean plus grass) was harvested at the beginning of soybean physiological maturity (R7 stage), which was when most pods on the main stem had reached mature colour. A forage harvester (Casale 180 harvester, São Carlos, São Paulo, Brazil) was used to collect the biomass material.

### 4.4. Macronutrient Accumulations in the Plants

The macronutrient accumulation in the plants of each cropping system was determined by multiplying the dry biomass of each species (soybean, Aruana Guinea grass, and Congo grass) by the concentration of each nutrient in the dry biomass. To estimate the biomass, all plants within a 1 metre row length of the usable area of each experimental plot were harvested, excluding the borders. The harvested green biomass was weighed and chopped. A representative subsample from each plot was then collected and dried in a forced-air circulation oven at 65 °C until constant weight to determine dry biomass. These dried subsamples were also used for macronutrient analysis.

The concentrations of N, P, K, Ca, Mg, and S in the plants were determined using the methods described by [42]. N was measured by the semi-micro Kjeldahl method after sulphuric acid digestion. P, K, Ca, Mg, and S were extracted by nitric-perchloric acid digestion. After extraction, P was determined by colorimetry, K by flame photometry, Ca and Mg by atomic absorption spectrophotometry, and S by turbidimetry with barium chloride.

### 4.5. Efficiency Indexes in the Cropping Systems

The physiological efficiency, N use efficiency, and efficiency of conversion of N to biomass (ECN) of soybean, Aruana Guinea grass, and Congo grass were calculated for each cropping system based on the methodology proposed by [42].

The physiological efficiency (PE, kg kg^−1^) was calculated as the difference between total dry biomass with and without N supply divided by the difference in the N accumulation with and without N supply (Equation (1)), which is as follows:

(1) $$\mathrm{PE}\;=\;\frac{\left({\mathrm{Biomass}}_{n}-{\mathrm{Biomass}}_{0}\right)}{\left({\mathrm{AcN}}_{n}-{\mathrm{AcN}}_{0}\right)}$$

where Biomass_n_ is dry biomass with N supply, Biomass_0_ is dry biomass without N supply, AcN_n_ is N accumulation with N supply, and AcN_0_ is N accumulation without N supply.

The nitrogen recovery efficiency (NRE) was calculated as the difference between AcN_n_ and AcN_0_ divided by the quantity of N supply ((N_applied) (Equation (2)), which is as follows:

(2) $$\mathrm{NRE}\;=\;\frac{\left({\mathrm{AcN}}_{n}-{\mathrm{AcN}}_{0}\right)}{\left(N\text{\_}applied\right)}$$

where AcN_n_ is N accumulation with N supply, AcN_0_ is N accumulation without N supply, and N supply is N_applied.

The N use efficiency (NUE, kg kg^−1^) was determined by multiplying the PE by NRE (Equation (3)), which is as follows:

(3) NUE = PE × NRE

where PE is the physiological efficiency and NRE is nitrogen recovery efficiency.

The ECN (kg kg^−1^) was calculated by dividing the total shoot dry biomass (Biomass_shoot, kg) by the N content in the shoot biomass (N_shoot, kg) (Equation (4)), which is as follows:

(4) $$\mathrm{ECN}\;=\;\frac{Biomass\text{\_}shoot}{N\text{\_}shoot}$$

where Biomass_shoot is total shoot dry biomass and N_shoot is N content in the shoot biomass.

### 4.6. Statistical Analysis

All data were analyzed using SAS software (Version SAS/STAT^®^ 9.2. I) [44]. For the first crop, an analysis of variance (ANOVA) was conducted, followed by a post hoc Tukey’s honest significant difference test (HSD) at a 5% significance level (*p* < 0.05). For the second cropping season, the data were analyzed using the General Linear Model (GLM) procedure in SAS at a 5% significance level (*p* < 0.05). Once significant interactions were identified, Tukey’s test (*p* < 0.05) was used to compare the means of each cropping system within each N rate, and regression analyses were used to examine the effects of N rates within the cropping systems. For main effects in isolation, cropping systems means were compared using Tukey’s test, and regression analyses were fitted to evaluate the effects of N rates.

## 5. Conclusions

Aruana Guinea grass intercropped with soybean increased the accumulation of N, P, K, and S in soybean crop. An inverse relationship was observed for potassium accumulation, with the highest K accumulation in soybean corresponding to the lowest K accumulation in Aruana Guinea grass. Nitrogen applied to the previous crop negatively affected the accumulation of P, K, and S in soybean monoculture. In contrast, N application altered the accumulation of N, P, Ca, Mg, and S in Aruana Guinea grass intercropped with soybean.

The maximum physiological efficiency of soybean in all cropping systems was directly related to the previous nitrogen supply. Soybean monoculture showed the lowest N use efficiency, whereas soybean–Congo grass intercropping resulted in the highest efficiency for converting N into biomass. Additionally, the high physiological efficiency, N use efficiency, and efficiency for converting N into biomass in Aruana Guinea grass highlighted its ability to recover residual N applied to the previous crop when intercropped with soybean.

Continued research on intercropping systems and macronutrient supply is necessary to improve long-term macronutrient use efficiency, particularly for cropping in degraded pasture areas.

## Figures and Tables

**Table 1:** Macronutrients accumulation (kg ha^−1^) in the soybean as function of the cropping systems and N rate applied in the maize crop.

Cropping Systems	N Rates (kg ha^−1^)				Means	F Test for Regression	
	0	50	100	150		Linear	Quadratic
N accumulation							
SM	4.36 ± 0.78 a	2.82 ± 0.78 a	3.96 ± 0.78 a	0.72 ± 0.78 a	2.96 ± 0.39 a	0.0686	0.1532
S + AGG	1.44 ± 0.78 a	1.95 ± 0.78 a	2.03 ± 0.78 a	2.49 ± 0.78 a	1.98 ± 0.39 a	0.2739	0.5620
S + CG	1.74 ± 0.78 a	4.81 ± 0.78 a	2.20 ± 0.78 a	2.67 ± 0.78 a	2.86 ± 0.39 a	0.9721	0.4703
Means	2.51 ± 0.45	3.20 ± 0.45	2.73 ± 0.45	1.96 ± 0.45		0.4110	0.3223
P accumulation							
SM	0.40 ± 0.07 a	0.28 ± 0.07 a	0.36 ± 0.07 a	0.07 ± 0.07 a	0.28 ± 0.04 a	0.0541	0.1175
S + AGG	0.13 ± 0.07 a	0.16 ± 0.07 a	0.18 ± 0.07 a	0.26 ± 0.07 a	0.18 ± 0.04 a	0.1536	0.3476
S + CG	0.15 ± 0.07 a	0.51 ± 0.07 a	0.21 ± 0.07 a	0.26 ± 0.07 a	0.28 ± 0.04 a	0.9599	0.3454
Means	0.23 ± 0.0 4	0.32 ± 0.04	0.25 ± 0.04	0.20 ± 0.04		0.5298	0.3600
K accumulation							
SM	2.76 ± 0.47 a	1.58 ± 0.47 a	2.13 ± 0.47 a	0.39 ± 0.47 a	1.71 ± 0.23 a	0.0510	0.1478
S + AGG	0.86 ± 0.47 a	0.99 ± 0.47 a	1.09 ± 0.47 a	1.40 ± 0.47 a	1.08 ± 0.23 a	0.3030	0.5826
S + CG	1.05 ± 0.47 a	3.15 ± 0.47 a	1.09 ± 0.47 a	1.87 ± 0.47 a	1.79 ± 0.23 a	0.8894	0.8099
Means	1.55 ± 0.27	1.91 ± 0.27	1.44 ± 0.27	1.22 ± 0.27		0.3565	0.4771
Ca accumulation							
SM	0.88 ± 0.19 a	0.64 ± 0.19 ab	0.99 ± 0.19 a	0.27 ± 0.19 a	0.70 ± 0.09 a	0.1889	0.2681
S + AGG	0.40 ± 0.19 a	0.36 ± 0.19 b	0.38 ± 0.19 a	0.61 ± 0.19 a	0.44 ± 0.09 a	0.3291	0.4257
S + CG	0.32 ± 0.19 a	1.49 ± 0.19 a	0.48 ± 0.19 a	0.55 ± 0.19 a	0.71 ± 0.09 a	0.8350	0.2569
Means	0.54 ± 0.11	0.83 ± 0.11	0.62 ± 0.11	0.48 ± 0.11		0.5667	0.2850
Mg accumulation							
SM	0.70 ± 0.12 a	0.42 ± 0.12 ab	0.69 ± 0.12 a	0.12 ± 0.12 a	0.48 ± 0.06 a	0.0776	0.1603
S + AGG	0.27 ± 0.12 a	0.30 ± 0.12 b	0.30 ± 0.12 a	0.43 ± 0.12 a	0.33 ± 0.06 a	0.3271	0.4650
S + CG	0.28 ± 0.12 a	0.90 ± 0.12 a	0.36 ± 0.12 a	0.47 ± 0.12 a	0.50 ± 0.06 a	0.9889	0.3974
Means	0.4179	0.5421	0.4471	0.3383		0.4345	0.3483
S accumulation							
SM	0.24 ± 0.04 a	0.16 ± 0.04 ab	0.19 ± 0.04 a	0.03 ± 0.04 a	0.16 ± 0.02 a	0.0191	0.0506
S + AGG	0.07 ± 0.04 a	0.10 ± 0.04 b	0.10 ± 0.04 a	0.13 ± 0.04 a	0.10 ± 0.02 a	0.2308	0.5010
S + CG	0.09 ± 0.04 a	0.28 ± 0.04 a	0.11 ± 0.04 a	0.16 ± 0.04 a	0.16 ± 0.02 a	0.8795	0.3868
Means	0.13 ± 0.02	0.18 ± 0.02	0.14 ± 0.02	0.11 ± 0.02		0.3522	0.2619

SM = soybean monoculture, S + AGG = soybean–Aruana Guinea grass intercropping, and S + CG = soybean–Congo grass intercropping. Means followed by different lowercase letter in the columns differ from each other according to Tukey’s test (*p* < 0.05).

**Table 2:** Macronutrients accumulation (kg ha^−1^) in the tropical grasses as function of the cropping systems and N rates applied to the previous crop.

Cropping Systems	N Rates (kg ha^−1^)				Means	F Test for Regression	
	0	50	100	150		Linear	Quadratic
N accumulation							
AGG	87.98 ± 10.86 a	56.30 ± 10.86 a	96.71 ± 10.86 a	130.71 ± 10.86 a	92.92 ± 5.43 a	0.0265	0.0067
CG	71.99 ± 10.86 a	105.47 ± 10.86 a	83.20 ± 10.86 a	93.30 ± 10.86 a	88.49 ± 5.43 a	0.4924	0.5484
Means	79.98 ± 7.68	80.89 ± 7.68	89.95 ± 7.68	112.00 ± 7.68		0.0269	0.3032
P accumulation							
AGG	8.06 ± 0.90 a	5.20 ± 0.90 b	8.81 ± 0.90 a	10.73 ± 0.90 a	8.20 ± 0.45 b	0.0403	0.0120
CG	7.67 ± 0.90 a	12.26 ± 0.90 a	9.99 ± 0.90 a	9.19 ± 0.90 a	9.78 ± 0.45 a	0.7209	0.0487
Means	7.87 ± 0.64	8.73 ± 0.64	9.40 ± 0.64	9.96 ± 0.64		0.1085	0.2785
K accumulation							
AGG	83.31 ± 8.16 a	61.58 ± 8.16 b	93.42 ± 8.16 a	108.04 ± 8.16 a	86.59 ± 4.08 a	0.0843	0.0862
CG	92.94 ± 8.16 a	113.74 ± 8.16 a	89.95 ± 8.16 a	95.95 ± 8.16 a	98.14 ± 4.08 a	0.6621	0.5633
Means	88.13 ± 5.77	87.66 ± 5.77	91.68 ± 5.77	101.99 ± 5.77		0.2023	0.3571
Ca accumulation							
AGG	21.66 ± 2.80 a	15.89 ± 2.80 b	22.50 ± 2.80a	28.45 ± 2.80 a	22.13 ± 1.40 a	0.0742	0.0332
CG	16.81 ± 2.80 a	33.35 ± 2.80 a	25.07 ± 2.80a	21.86 ± 2.80 a	24.27 ± 1.40 a	0.7439	0.0263
Means	19.23 ± 1.98	24.62 ± 1.98	23.79 ± 1.98	25.16 ± 1.98		0.1793	0.3183
Mg accumulation							
AGG	28.18 ± 3.51 a	20.26 ± 3.51 b	28.38 ± 3.51 a	38.00 ± 3.51 a	28.70 ± 1.75 a	0.0862	0.0328
CG	23.94 ± 3.51 a	37.41 ± 3.51 a	31.47 ± 3.51 a	28.34 ± 3.51 a	30.29 ± 1.75 a	0.7239	0.1553
Means	26.06 ± 2.48	28.84 ± 2.48	29.92 ± 2.48	33.17 ± 2.48		0.1241	0.3120
S accumulation							
AGG	8.93 ± 1.33 a	4.89 ± 1.33 b	7.25 ± 1.33 a	7.72 ± 1.33 a	7.20 ± 0.66 a	0.7907	0.0281
CG	6.12 ± 1.33 a	12.27 ± 1.33 a	8.33 ± 1.33 a	8.27 ± 1.33 a	8.75 ± 0.66 a	0.8023	0.3631
Means	7.53 ± 0.94 a	8.58 ± 0.94 a	7.79 ± 0.94 a	7.99 ± 0.94 a		0.9108	0.9377

AGG = Aruana Guinea, and CG = Congo grass. Means followed by different capital letter in the columns differ from each other according to Tukey’s test (*p* < 0.05). Means followed by different lowercase letters in the lines differ from each other according to Tukey’s test (*p* < 0.05).

**Table 3:** Physiological efficiency, N use efficiency, and efficiency of conversion of N to biomass of the soybean as a function of the cropping systems and N rates applied to the previous.

Cropping System	N Rates (kg ha^−1^)					F-Test for Regression	
	0	50	100	150	Means	Linear	Quadratic
Physiological efficiency (kg kg^−1^)							
SM	-	33.90 ± 5.08 a	26.15 ± 5.08 a	25.86 ± 5.08 a	21.48 ± 2.54 a	0.0399	0.0031
S + AGG	-	23.64 ± 5.08 a	33.29 ± 5.08 a	35.98 ± 5.08 a	23.23 ± 2.54 a	0.0026	0.0045
S + CG	-	35.98 ± 5.08 a	26.67 ± 5.08 a	35.09 ± 5.08 a	24.44 ± 2.54 a	0.0113	0.0070
Means	-	31.17 ± 2.93	28.70 ± 2.93	32.31 ± 2.93		0.0001	0.0001
N use efficiency (kg kg^−1^)							
SM	-	−0.76 ± 0.43 b	0.01 ± 0.43 a	−0.61 ± 0.43 a	−0.34 ± 0.22 b	0.7064	0.9283
S + AGG	-	0.13 ± 0.43 a	0.13 ± 0.43 a	0.23 ± 0.43 a	0.12 ± 0.22 ab	0.4719	0.7784
S + CG	-	2.37 ± 0.43 a	0.13 ± 0.43 a	0.21 ± 0.43 a	0.68 ± 0.22 a	0.6297	0.2492
Means	-	0.58 ± 0.25	0.09 ± 0.25	−0.06 a ± 0.25		0.6644	0.5107
Efficiency of conversion of N to biomass (kg kg^−1^)							
SM	3.12 ± 0.6 a	2.20 ± 0.66 b	3.57 ± 0.66 a	0.83 ± 0.66 a	2.43 ± 0.33 a	0.1794	0.2469
S + AGG	1.13 ± 0.66 a	1.13 ± 0.66 b	1.40 ± 0.66 a	2.33 ± 0.66 a	1.50 ± 0.33 a	0.1629	0.2887
S + CG	1.30 ± 0.66 a	5.82 ± 0.66 a	1.71 ± 0.66 a	2.40 ± 0.66 a	2.80 ± 0.33 a	0.8783	0.2918
means	1.85 ± 0.38	3.05 ± 0.38	2.22 ± 0.38	1.85 ± 0.38		0.7403	0.3481

SM = soybean monoculture, S + AGG = soybean–Aruana Guinea grass intercropping, and S + CG = soybean–Congo grass intercropping. Means followed by different lowercase letters in the columns differ from each other according to Tukey’s test (*p* < 0.05). -: There is no physiological efficiency or nitrogen use efficiency in the absence of nitrogen supply.

**Table 4:** Physiological efficiency, N use efficiency, and efficiency of conversion of N to biomass of grasses as a function of the cropping systems and N rates applied to the previous crop.

Cropping System	N Rates (kg ha^−1^)					F-Test for Regression	
	0	50	100	150	Means	Linear	Quadratic
Physiological efficiency (kg kg^−1^)							
AGG	-	39.88 ± 46.03 a	83.04 ± 46.03 a	104.29 ± 46.03 a	56.80 ± 23.02 a	0.0091	0.0359
CG	-	54.58 ± 46.03 a	189.81 ± 46.03 a	55.84 ± 46.03 a	75.06 ± 23.02 a	0.3651	0.2907
Means	-	47.23 ± 32.55	136.42 ± 32.55	80.07 ± 32.55		0.0579	0.0653
N use efficiency (kg kg^−1^)							
AGG	-	−23.32 ± 5.67 b	8.79 ± 5.67 a	15.06 ± 5.67 a	0.13 ± 2.83 b	0.0637	0.0406
CG	-	28.93 ± 5.67 a	9.95 ± 5.67 a	4.75 ± 5.67 a	10.91 ± 2.83 a	0.9033	0.0409
Means	-	2.80 ± 4.00	9.37 ± 4.00	9.90 ± 4.00		0.2148	0.8626
Efficiency of conversion of N to biomass (kg kg^−1^)							
AGG	299.16 ± 42.26 a	268.99 ± 42.26 a	374.51 ± 42.26 a	427.43 ± 42.26 a	342.52 ± 21.13 a	0.0442	0.1023
CG	270.02 ± 42.26 a	328.56 ± 42.26 a	351.12 ± 42.26 a	286.20 ± 42.26 a	308.97 ± 21.13 a	0.7370	0.3935
Means	284.59 ± 29.89	298.77 ± 29.89	362.81 ± 29.89	356.81 ± 29.89		0.0785	0.2096

S + AGG = Aruana Guinea grass, and CG = Congo grass intercropping. Means followed by different lowercase letters in the columns differ from each other according to Tukey’s test (*p* < 0.05). -: There is no physiological efficiency or nitrogen use efficiency in the absence of nitrogen supply.

## Data Availability

The data that support this study are available in the article.
